# Recessive mutations in *MSTO1* cause mitochondrial dynamics impairment, leading to myopathy and ataxia

**DOI:** 10.1002/humu.23262

**Published:** 2017-06-06

**Authors:** Alessia Nasca, Chiara Scotton, Irina Zaharieva, Marcella Neri, Rita Selvatici, Olafur Thor Magnusson, Aniko Gal, David Weaver, Rachele Rossi, Annarita Armaroli, Marika Pane, Rahul Phadke, Anna Sarkozy, Francesco Muntoni, Imelda Hughes, Antonella Cecconi, György Hajnóczky, Alice Donati, Eugenio Mercuri, Massimo Zeviani, Alessandra Ferlini, Daniele Ghezzi

**Affiliations:** ^1^ Molecular Neurogenetics Unit Foundation IRCCS Neurological Institute Besta Milan Italy; ^2^ Medical Genetics Unit Department of Medical Sciences University of Ferrara Ferrara Italy; ^3^ Dubowitz Neuromuscular Centre UCL Great Ormond Street Hospital London UK; ^4^ deCODE Genetics Reykjavik Iceland; ^5^ MitoCare Center for Mitochondrial Imaging Research and Diagnostics Department of Pathology Anatomy and Cell Biology Thomas Jefferson University Philadelphia Pennsylvania; ^6^ Institute of Genomic Medicine and Rare Disorders Semmelweis University Budapest Hungary; ^7^ Neuropsichiatry Unit Catholic University Policlinico Gemelli Rome Italy; ^8^ Royal Manchester Children's Hospital Manchester UK; ^9^ Pediatrics Medical Genetics Hospital S. Maria Annunziata Bagno a Ripoli Florence Italy; ^10^ Unit of Metabolic and Muscular Diseases Meyer Children Hospital Florence Italy; ^11^ Mitochondrial Biology Unit ‐ MRC Cambridge UK

**Keywords:** ataxia, mitochondrial dynamics, MSTO1, myopathy, skeletal abnormalities

## Abstract

We report here the first families carrying recessive variants in the *MSTO1* gene: compound heterozygous mutations were identified in two sisters and in an unrelated singleton case, who presented a multisystem complex phenotype mainly characterized by myopathy and cerebellar ataxia. Human MSTO1 is a poorly studied protein, suggested to have mitochondrial localization and to regulate morphology and distribution of mitochondria. As for other mutations affecting genes involved in mitochondrial dynamics, no biochemical defects typical of mitochondrial disorders were reported. Studies in patients’ fibroblasts revealed that MSTO1 protein levels were strongly reduced, the mitochondrial network was fragmented, and the fusion events among mitochondria were decreased, confirming the deleterious effect of the identified variants and the role of MSTO1 in modulating mitochondrial dynamics. We also found that MSTO1 is mainly a cytosolic protein. These findings indicate recessive mutations in *MSTO1* as a new cause for inherited neuromuscular disorders with multisystem features.

Mitochondria are highly dynamic organelles, which undergo constant fusion and fission events, and cristae remodeling. Mitochondrial dynamics influences several homeostatic and execution pathways involving the organelle and other compartments of the cell, and is in turn controlled by intraorganellar and extraorganellar metabolic requirements and signals, including the availability of nutrients, cell cycle, and calcium homeostasis. Mitochondrial fusion allows the exchange of damaged mitochondrial DNA (mtDNA) and proteins between impaired and “healthy” mitochondria, whereas mitochondrial fission facilitates their redistribution inside the cell (Okamoto & Shaw, 2005) and the isolation of faulty mitochondria, which can then be eliminated by mitophagy. Fragmentation of the mitochondrial network is also observed just before apoptosis (Munoz‐Pinedo et al., 2006). Mitochondrial dynamics is mainly controlled by a group of proteins belonging to the dynamin superfamily of GTPases. The typical structure of dynamin‐related proteins consists of a large amino‐terminal GTPase domain, a middle domain, and a GTPase effector domain (Praefcke & McMahon, 2004). DNM1L (dynamin‐1‐like, MIM# 603850) is a cytosolic GTPase that localizes to the outer mitochondrial membrane and carries out mitochondrial fission. Other members of the dynamin family are involved in mitochondrial fusion: mitofusins (MFN1, MIM# 608506, and MFN2, MIM# 608507) localize to and operate the fusion of the mitochondrial outer membrane (Chen et al., 2003), whereas OPA1 (MIM# 605290) is a multitasking protein of the inner membrane, where it promotes fusion, cristae remodeling, and sealing of cristae junctions (Alexander et al., 2000; Delettre et al., 2000). Very recently, dynamin‐2 (DNM2, MIM# 602378) has been reported to be a fundamental component of the mitochondrial fission machinery (Lee, Westrate, Wu, Page, & Voeltz, 2016).

The human gene *MSTO1* encodes the ortholog of misato of *D. melanogaster*. Misato proteins are conserved from yeast to human. Their function is still controversial, but they share regions homologous to a GTPase subfamily (Miklos, Yamamoto, Burns, & Maleszka, 1997) that includes tubulin and bacterial FtsZ, regulating segregation of chromosomes, and fission of both chloroplasts and mitochondria. Human MSTO1 is ubiquitously expressed (Kimura & Okano, 2007) and was reported to localize mainly to mitochondria. RNAi‐mediated MSTO1 knockdown causes mitochondrial fragmentation, whereas overexpression of recombinant MSTO1 induces aggregation of mitochondria at the perinuclear region (Kimura & Okano, 2007).

Several mutations in genes‐encoding factors mediating mitochondrial fission or fusion have been associated with diverse human genetic diseases, with predominant neurological involvement. For instance, mutations in *MFN2, OPA1, DNM1L*, and *MFF* (encoding mitochondrial fission factor) cause Charcot–Marie–Tooth disease type 2A (MIM# 609260), dominant optic atrophy (MIM# 165500), and severe infantile encephalomyopathies (MIM# 614388; 617086), respectively. Here, we report a family of four with two sisters, presenting with severe congenital myopathy and cerebellar ataxia associated with skeletal abnormalities and mild mental retardation as corollary symptoms of a multisystem disease. Whole‐exome sequencing (WES) analysis identified a compound heterozygous genotype of two missense mutations in *MSTO1*. Functional characterization of mutant cells demonstrated profound MSTO1 reduction in patients’ fibroblasts. Different compound heterozygous variants in *MSTO1* were found by WES also in a singleton case presenting a dystrophic myopathy and cerebellar ataxia. Our results demonstrate that *MSTO1* is a novel disease gene causing a mitochondrial morphology defect and leading to a muscular recessive disease (ranging from congenital myopathy to muscular dystrophy) with multisystem involvement.

Detailed methods for biochemical assays, molecular genetics, structural and protein analyses, fluorescence microscopy, and functional studies in cells are reported in Supp. Information.

Informed consent for genetic and biochemical studies was obtained from the parents of patients, in agreement with the Declaration of Helsinki and approved by the Ethical Committees of the Meyer Children Hospital, Florence, Italy, and of the Health Research Authority, NRES Committee East of England – Hatfield.

Patient A1 is the first daughter of unrelated parents (II‐1, family A; Fig. 1A). Family history is negative for neurological and skeletal disorders. Pregnancy was uneventful, but was interrupted prematurely by caesarean delivery due to acute fetal distress. The child showed neonatal distress (Apgar 5–7), but her psychomotor development was reported as normal in the first months after birth. However, from 8 to 9 months of age, severe growth and motor delay became progressively obvious. A brain MRI at 18 months revealed severe hypotrophy of cerebellar vermis, with an enlarged cisterna magna, and hyperintense signals in the supratentorial periventricular and posterior white matter. At 5 years of age, the patient showed severe growth impairment (<3^rd^ percentile for both weight and height), had fine tremors and no autonomous walk, but normal muscle tone and normal cognitive development. At 7 years of age, a brain MRI disclosed global cerebellar hypotrophy, with reduction of the N‐acetyl aspartate peak at proton magnetic resonance spectroscopy ([^1^H^+^]‐MRS), and abnormal signal in the supratentorial peritrigonal white matter. These findings were confirmed at 16 years of age (Fig. 1B and C). Ophthalmological examination revealed the presence of pigmentary retinopathy with papillary pallor, associated with concentric restriction of the visual field but no abnormality of visual acuity. Electromyography (EMG) examination revealed a myopathic pattern, and a muscle biopsy confirmed the presence of myopathic features with high variability in muscle fiber diameter hypotrophic, polygonal fibers and hypertrophic, round fibers, and scattered fibers with intracytoplasmic microvacuolizations. No oxidative histochemical defect in mitochondrial enzymes (COX, SDH) was observed. Plasma CK was elevated (≈1,200 U/L, n.v. <250), and reduced citrullin levels were consistently found in plasma. The patient showed severe asymmetry of the chest, with enlargement of the right hemithorax, pectus excavatum, and marked scoliosis (Fig. 1D). She was able to stand but could not walk autonomously. Her face is peculiar with thick hair and a high‐arched palate. Biochemical assays of muscle homogenate showed no abnormality of the mitochondrial respiratory chain, but citrate synthase activity, an index of mitochondrial mass, was markedly reduced (36 nmol/min/mg, n.v. 80–210; Fig. 1E). mtDNA amount was reduced to 27% of the mean control value (Fig. 1F). The following genes were previously excluded by Sanger sequencing: *SPG7*, *FKTN, POMT1, POMT2, LARGE, POMGNT1*, and *SIL1*. The presence of pathogenic mtDNA mutations was also ruled out. The patient is now 17 years old, in stable condition, with severely impaired motor function, severe skeletal abnormalities, but no cognitive deficiency.

**Figure 1:**
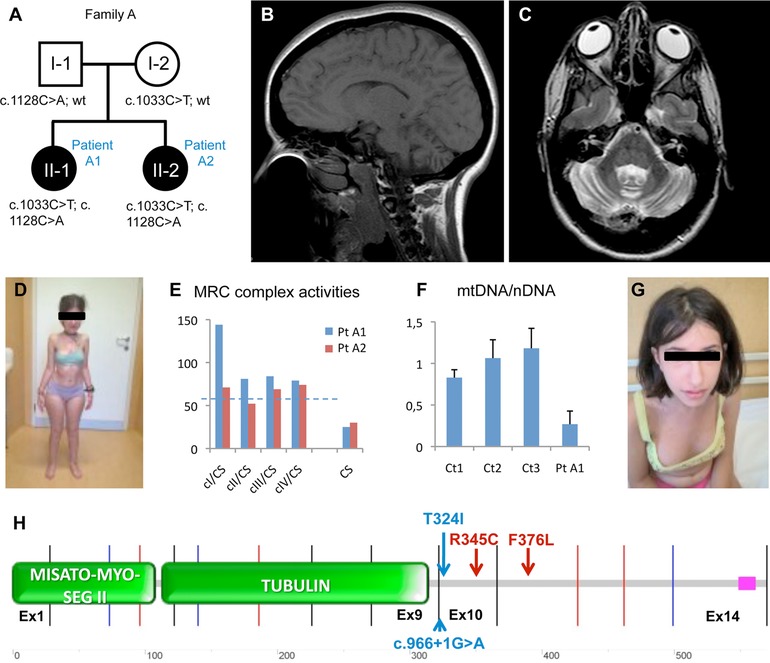
Clinical, biochemical, and genetic features of the *MSTO1* mutant patients A1 and A2. **A**: Pedigree of the family A with the identified *MSTO1* variants. Black symbols indicate the affected siblings. **B** and **C**: Brain MRI (**B**: sagittal; **C**: coronal images) of the patient A1, taken at 16 years of age, showing cerebellar hypotrophy. **D**: Skeletal abnormalities of patient A1 (17 years of age), with marked scoliosis and severe asymmetry of the chest and pectus excavatum. Consent to publish anonymized photos was obtained from patients’ parents. **E**: Activities of the mitochondrial respiratory chain (MRC) complexes (cI, cII, cIII, cIV) in muscle homogenates from patients A1 and A2, reported as percentages of the controls mean. The specific activities were normalized for citrate synthase (CS) activity. The dotted line represents the minimum value of the control range. **F**: Quantification of mtDNA amount in muscle from patient A1 and 3 controls (Ct1‐3). The bars represent the amount of mtDNA normalized to nuclear DNA (nDNA), compared with the mean value of controls (= 1). Data are represented as mean ± SD of four independent experiments. **G**: Dysmorphic traits of patient A2: triangular face, with sunken eyes, severe asymmetry of the chest and pectus excavatum. **H**: Schematic representation of the MSTO1 protein with main functional domains and localization of the identified *MSTO1* mutations in family A (red variants) and B (blue variants)

Patient A2, the younger sister of patient A1 (II‐2, family A; Fig. 1A), was born by cesarean section after an uneventful pregnancy. Her clinical course has been similar to that of patient A1, including the presence of cerebellar hypotrophy with no autonomous walk, adiadochokinesia, dysmetry and fine tremors; she shows muscle weakness, pes cavus, and reduced deep tendon reflexes. An EMG revealed a predominantly myopathic pattern, associated with high plasma levels of CK (1,872 U/L) and low plasma levels of citrullin (14 μM, n.v. 17–53). Her cognitive development has been normal but, like her affected sister, she showed severe growth impairment since 8–9 months of age; facial (Fig. 1G) and hair abnormalities and asymmetry of the thorax were similar to those of her sister, but she showed no organic aciduria and normal acylcarnitines in plasma and urine. Ophthalmological examination revealed bilateral papillary pallor, but pigmentary retinopathy, reduced visual acuity, and nystagmus were absent. A muscle biopsy showed a myopathy with dystrophic features, with vacuolization of scattered muscle fibers, increased perimysial‐ and endomysial connective tissue, and partial reduction of COX histoenzymatic reaction. Biochemical assays revealed partial complex II deficiency (52% residual activity) and severely reduced citrate synthase activity (44 nmol/min/mg; Fig. 1E). No residual muscle was available for further analyses. The patient is now 13 years old, in a stable condition.

Patient B is a 7‐year‐old child of British Caucasian descent, born to nonconsanguineous parents (family B); he has a healthy older sister. He presented at around 1 year of age with reluctance to stand and walk unsupported. Subsequently, he showed difficulty walking long distances, getting up from the floor, and frequent falls. Speech acquisition was delayed. Gradual improvement in motor activities was then noted and, at the age of 3.5 years, he was able to walk half a mile, although with frequent falls, and he acquired the ability to walk upstairs. Gowers’ time from lying was 6.4 sec. At the age of 6 years, he showed further improvement, showing less falls and a Gower's time of 3.6 sec. Weakness was proximal more than distal, affecting lower limbs (MRC power grade 3–4/5) more than upper (MRC power grade 4/5), with the exception of the peroneal muscles that were MRC power grade 3+/5. He presents mild end of range tightness of Achilles tendons but no other joint contractures. Deep tendon reflexes were present and symmetric; Babinski was negative. A degree of poor coordination, especially of the left hand, was noticeable from the age of 3 years. At the age of 6 years, he showed a fine intentional tremor bilaterally at finger‐nose test, dysdiachokinesia and difficulties with rapid hands repeated movements but no nystagmus. He shows no cognitive involvement but presents problems related to speech articulation. He has no cardiac or respiratory involvement, and forced vital capacity was 91% of predicted values. Ophthalmological examination was normal. An EMG/NCS showed changes compatible with a myopathic process. Brain MRI at the age of 3.5 years showed hypoplasia of cerebellar vermis and hemispheres (Supp. Fig. S1). These findings remained static at a follow up of 2 years. Serum CK was 4,520 IU/L and serum lactate and transferrin were normal. Right thigh muscle biopsy at 2 years showed dystrophic changes with nonrimmed vacuoles. Oxidative histochemistry (COX, SDH) did not show COX‐ or SDH‐deficient activities. Ultrastructural examination showed striking vacuolar degeneration of mitochondria (Supp. Fig. S2).

Mutations in known genes causative of muscular dystrophies and vacuolar myopathies were excluded first by a gene panel analysis, and direct Sanger sequencing of a number of limb girdle muscular dystrophy genes was also performed. Further search of mutations in less common neuromuscular genes was excluded by WES data analysis. Variants were first prioritized according to a homozygous‐recessive model, but no variants were found. Therefore, variants were prioritized according to the presence of compound heterozygosity based on recessive inheritance and on the nonconsanguinity of the parents. In the two affected siblings of family A (A1 and A2), two novel missense mutations were identified in *MSTO1*, a gene regulating mitochondrial distribution and morphology, not previously associated with human diseases (Supp. Fig. S3) (GenBank accession no. NM_018116.3): c.1033C>T; p.R345C and c.1128C>A; p.F376L (Fig. 1H). The R345 and F376 are highly conserved residues (Supp. Fig. S4A), and the corresponding variants have never been reported in publicly available databases [http://www.ncbi.nlm.nih.gov/SNP; http://browser.1000genomes.org/index.html; http://exac.broadinstitute.org; ], excepting 1 out of >120,000 alleles with the c.1033C>T (rs150075701) in ExAC database. Sanger sequencing in the probands and their parents confirmed the presence of the (maternal) c.1033C>T and the (paternal) c.1128C>A variants in both patients (A1 and A2), as expected for an autosomal‐recessive nonconsanguineous inheritance (Supp. Fig. S4A; Fig. 1A). Following this finding, we searched for additional patients with pathogenic variants in *MSTO1* in the WES datasets from congenital myopathy and congenital muscular dystrophy patients analysed within the NeurOmics project. In patient B, we identified two heterozygous *MSTO1* mutations: c.971C>T; p.T324I and a nucleotide change c.966+1G>A affecting the consensus splice site of exon 9 (Fig. 1H). Sanger sequencing confirmed the segregation of the mutations with the disease within the family (Supp. Fig. S5). Both varaints are present in the ExAC database at very low frequency compatible with rare recessive disorder (allele frequency < 0.00004).

In order to evaluate the effect of the identified *MSTO1* variants on transcript and protein, we studied available specimens from the patients. For A1 and A2, we used skin fibroblasts: quantitative PCR analysis of the retrotranscribed *MSTO1* cDNA showed partial reduction of transcript amount (Supp. Fig. S6A) in both cell lines, but no aberrant species were observed. Notably, the total amount of MSTO1 protein detected by western blot (WB) analysis was drastically reduced in patients’ fibroblasts compared with controls (∼15% residual amount; Fig. 2A); a strong reduction in MSTO1 was also observed in immortalized fibroblasts from A1 (Supp. Fig. S6B). These findings confirmed the deleterious effect of these two *MSTO1* variants, probably affecting the stability of the protein. For patient B, we analyzed skeletal muscle: transcript analysis of cDNA obtained from retrotranscribed RNA showed the presence of an aberrant transcript that was found to lack exon 9. This finding confirmed the deleterious effect of the c.966+1G>A on splicing (Supp. Fig. S7). Then, we tested the antibody on total lysate, but no immunoreactive signal was observed at the expected molecular weight even in control samples (Supp. Fig. S7), suggesting that the antibody does not work on muscle homogenate.

**Figure 2:**
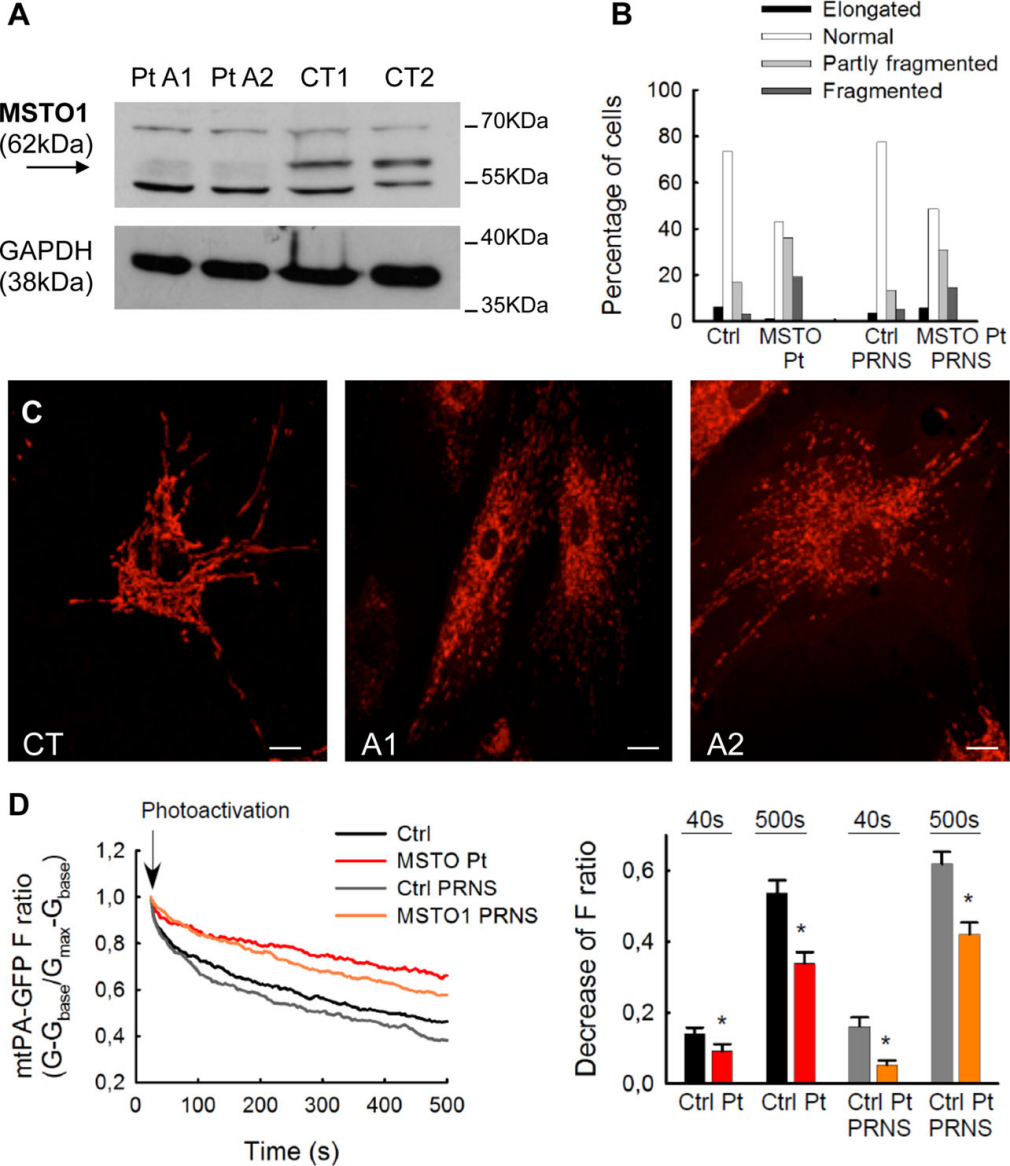
Characterization of fibroblast cell lines. **A**: MSTO1 protein amount in patients’ (A1 and A2) and control (CT1 and CT2) fibroblasts, obtained using an anti‐MSTO1 antibody. An anti‐GAPDH antibody was used as a loading control. **B**: Mitochondrial morphology, assessed in primary and pRNS1‐immortalized fibroblasts from patient A2 and controls (Pt and CT, iPt and iCT, respectively), was scored as follows: “Fragmented”, mainly small and round; “Partly fragmented”, intermediate, mixture of round and shorter tubulated; “Normal”, tubulated, long and higher interconnectivity; “Elongated”, very long, tubulated. The percentage of cells with indicated mitochondrial morphologies was determined as a percentage of the total number of MitoTracker Green loaded cells counted (number of cells: CT = 145, Pt = 160; iCT = 191; iPt = 220). **C**: Representative images of mitochondrial morphology (obtained with MitoTracker red), showing the filamentous mitochondrial network of fibroblasts from a control (CT), and the fragmented network in patients’ (A1 and A2) cells, grown in glucose medium. Scale bar: 25 μm. **D**: Mitochondrial continuity in primary and pRNS1‐immortalized fibroblasts. The time course of the F ratio of mtPA‐GFP (G‐G_base_/G_max_‐G_base_) for the region of photoactivation (RPA) (left); the decay of the fluorescence ratio in the RPA at 500 sec (right). (Number of imaged cells: CT = 20; Pt = 21; iCT = 23; iPt = 25; from three experiments per each cells). Stars indicate significant differences (*P*<0.05)

Given the predicted role of MSTO1 in regulating mitochondrial morphology (Kimura & Okano, 2007), we evaluated the functional consequences of the *MSTO1* variants by assessing the mitochondrial network in patients’ fibroblasts. Fibroblasts grown in either standard glucose or galactose medium were stained with a mitochondrial dye (Mitotracker) and examined by fluorescence microscopy. Patients’ cells showed a clear fragmentation of the mitochondrial network compared with controls. Altered mitochondria were also more abundant in immortalized fibroblasts from patient A1 than from controls (Fig. 2B and C). To evaluate whether the fragmented mitochondrial morphology in patient and control fibroblasts can result from suppressed mitochondrial fusion activity, cells were cotransfected with a mitochondrial marker (mtDsRed) and a mitochondrial photoactivatable GFP (mtPA‐GFP). The diffusion of the photo‐activated mtPA‐GFP was reduced in patients’ cells, indicating a decrease in the combined activity of mitochondrial network formation, mitochondrial fusion, and mitochondrial movements (Fig. 2D). The fusion events, validated by reciprocal spreading of mtPA‐GFP and mtDsRed, were fewer and lasted slightly longer in patients’ cells than in controls (Supp. Fig. S8). Thus, suppression of mitochondrial fusion is likely a cause of the fragmented mitochondrial morphology in *MSTO1* patients. Since some of the proteins involved in mitochondrial dynamics (e.g., DNM1L, MFF) are also involved in peroxisomal morphology, we did check this aspect by immunofluorescence using an antiperoxisomal antibody (PMP70): in patients’ cells, we observed a normal morphology and distribution of peroxisomes that had only a slightly, not significantly, reduced mean area compared with control cells (Supp. Fig. S9A and B).

In accordance with the reduced mtDNA content observed in muscle from A1, the amount of mtDNA in total DNA extracted from fibroblasts, assessed by quantitative PCR, was also lower in the patients than in controls (39% and 68% of the mean control value for A1 and A2, respectively); nevertheless, by Picogreen staining, we did not observe any evident alteration (e.g., perinuclear clustering) in mitochondrial nucleoid distribution (Supp. Fig. S9C). Respiratory capacity, assessed by SeaHorse micro‐oxygraphy, as well as citrate synthase activity, were normal in patients’ fibroblasts (not shown).

MSTO1 was previously suggested to be a mitochondrial protein, localized in the outer membrane (Kimura & Okano, 2007), but several bioinformatics tools for prediction of the subcellular localization indicated MSTO1 as a cytosolic protein, not targeted to mitochondria (Supp. Table S1). Moreover, in our WB experiments, the intensity of the anti‐MSTO1 immunoreactive band was lower in mitochondrial‐enriched preparations compared with total lysates; hence, we decided to investigate the localization of MSTO1 by WB immunodetection on HeLa cell subfractions. This analysis revealed that MSTO1 was present in the 13,000*g* supernatant, corresponding to the postmitochondrial fraction, but not in the mitochondria‐containing 13,000*g* pellet. Ultracentrifugation of the postmitochondrial supernatant at 100,000*g* indicated that MSTO1 is a soluble protein present in the 100,000*g* (cytosolic) supernatant but absent in the corresponding (microsomal) pellet (Supp. Fig. S10). Notably, the distribution of MSTO1 upon differential centrifugation is similar to that of DNM1L (also known as DRP1). Since the antibody against MSTO1 was not suitable for immunofluorescence studies, we expressed a HA‐tagged MSTO1 protein (MSTO1‐HA) in HeLa cells. The HA signal was distributed throughout the cytoplasm with only partial colocalization with a mitochondrial marker, whereas no signal was detected in the nucleus (Supp. Fig. S10), thus confirming the results of the immunoblot experiments. The same pattern was observed also in COS7 cells transfected with MSTO1‐HA (Supp. Fig. S11).

Next, we tested whether the expression of wild‐type MSTO1 could recover the fragmented mitochondrial network observed in mutant fibroblasts. To avoid artifacts caused by fixation, we analyzed the effect of *MSTO1* expression on mitochondria in live cells. MSTO1 was cotransfected with a GFP targeted to mitochondria (mtGFP) in order to easily identify cells expressing the exogenous proteins (Supp. Fig. S12). After transient overexpression of wild‐type MSTO1 in mutant and control fibroblasts, we observed that the mitochondrial network was unaffected at 24 hr, but at 48 hr, most of the cells with green signal collapsed, detached from the plate or floating in the medium; only a few cells were still adherent but showed fragmentation and perinuclear aggregation of mitochondria, both in patients and control cells (Supp. Fig. S13). At 72 hr, no cells with green signal were found. Contrariwise, the single transfection of mtGFP had no major effect on the viability of fibroblasts and on the mitochondrial network even after >96 hr (not shown). These findings suggest that the overexpression of MSTO1 is deleterious, as already reported by Kimura and Okano (2007), who observed reduced vitality and perinuclear clusters of mitochondria after overexpression of MSTO1‐GFP in COS7 cells.

The WES analysis in our families with myopathy and ataxia revealed that mutations of *MSTO1* can be a new genetic cause for rare human disease, impairing the mitochondrial dynamic maintenance circuit. The fragmented mitochondrial network observed in mutant cells and vacuolar degeneration of mitochondria are in agreement with the proposed role of MSTO1 in mitodynamics. In fact, RNAi‐driven *MSTO1* depletion leads to mitochondrial fragmentation, whereas overexpression of human EGFP‐tagged MSTO1 causes alterations in mitochondrial morphology and aggregation of mitochondria in the perinuclear region (Kimura & Okano, 2007). This was supported also by evidence from studies on *S. cerevisiae*, where expression of *DML1*, the bona fide yeast ortholog of MSTO1, leads to mitochondrial dispersion and abnormalities in cell morphology (Gurvitz, Hartig, Ruis, Hamilton, & de Couet, 2002). Taken together, these findings suggest that MSTO1 is either a profusion protein, which promotes the formation of the tubular network, or an antifission factor, which inhibits the activity of profission proteins such as DNM1L. Our data indicate that MSTO1 is largely cytosolic: this result is in contrast with those of Kimura and Okano (2007), who reported a loose association with the mitochondrial outer membrane. However, it is possible that MSTO1 may have the same behavior of DNM1L, which is a cytosolic protein that translocates to the outer membrane to carry out mitochondrial fission (Bleazard et al., 1999; Smirnova, Griparic, Shurland, & van der Bliek, 2001).

Given its homology with eukaryotic tubulin and prokaryotic FtsZ (Gurvitz et al., 2002; Miklos et al., 1997), MSTO1 was initially proposed to regulate chromosome segregation and cell division; accordingly, Misato mutations in *D. melanogaster* were associated with irregular chromosome segregation during mitosis (Miklos et al., 1997). Some clinical features in our patients A1 and A2, such as skeletal developmental abnormalities (e.g., pectus excavatum) and mild dysmorphic features do suggest a possible involvement of nuclear chromosomal DNA, whereas other abnormalities, such as cerebellar hypotrophy, leukodystrophy, and myopathic/dystrophic changes, possibly leading to additional skeletal deformities such as scoliosis, are often associated with mitochondrial impairment. However, considering that the larvae of *MSTO1* mutant flies showed severe morphological abnormalities and early death, the phenotype presented by our patients seems milder, suggesting a hypomorphic effect of the identified *MSTO1* variants. Another possibility is that the function of the protein may diverge between arthropoda and humans.

Most of the patients with mutations in genes related to mitodynamics presented a neurological phenotype; our *MSTO1*‐mutant subjects have indeed a myopathy associated with cerebellar ataxia, underlined by hypotrophy of the cerebellar vermis; the sisters A1 and A2 also present a multisystem condition with developmental delay, particularly motor impairment with ataxia and dysmorphisms.

Our biochemical analyses were not indicative of a mitochondrial disorder: patients showed normal lactate levels, and hardly any defect of MRC complex activities or cell respiration, as frequently seen in other defects of mitodynamics, such as *OPA1* (Mayorov et al., 2008) and *DNM1L* mutations (Nasca et al., 2016). However, we detected markedly low activity of citrate synthase and reduced mtDNA amount, suggesting a reduction in mitochondrial mass. Although these alterations can be due to dystrophic changes in the muscle biopsies, the low mtDNA content observed in patients’ fibroblasts supports the previous hypothesis. Interestingly, reduced plasma citrullin was found in both siblings A1 and A2: hypocitrullinemia was reported in some inherited disorders affecting mitochondrial function and was suggested as a biomarker of oxidative stress (Atkuri et al., 2009).

Our study confirms the proposed role for MSTO1 in regulating mitochondrial morphology, favoring fusion versus fission events, despite its mainly cytoplasmic localization. We showed that compound heterozygous recessive mutations in *MSTO1* lead to impairment of mitochondrial dynamics, and are associated, in our affected patients, with a disease characterized by neurological, muscular, and sometimes skeletal involvement. Notably, a family with an autosomal‐dominant mutation in *MSTO1* was recently identified (Gal et al., 2016), with the affected subjects presenting symptoms partly overlapping those of our patients, for example, myopathy, ataxia, skeletal alterations, in addition to psychotic features, for example, schizophrenia or autism, which are not present in our cases. Certainly, investigation on further patients is required to better define the mode of inheritance and the clinical spectrum of *MSTO1*‐related disease.

## Supporting information

ONLINE SUPPORTING INFORMATION Recessive mutations in *MSTO1* cause mitochondrial dynamics impairment, leading to myopathy and ataxia
