# Supplementary material for: Recessive mutations in MSTO1 cause mitochondrial dynamics impairment, leading to myopathy and ataxia
Source: Hum Mutat. 2017 Jun 6;38(8):970–7. doi: 10.1002/humu.23262 (PMC5575512; doi:10.1002/humu.23262)
Supplement: Supplementary file 1 — ONLINE SUPPORTING INFORMATION Recessive mutations in MSTO1 cause mitochondrial dynamics impairment, leading to myopathy and ataxia [file HUMU-38-970-s001.pdf]

ONLINE SUPPORTING INFORMATION

Recessive mutations in *MSTO1* cause mitochondrial dynamics impairment, leading to myopathy and ataxia

Alessia Nasca[1,]*, Chiara Scotton[2,]*, Irina Zaharieva[3], Marcella Neri[2], Rita Selvatici[2], Olafur Thor Magnusson[4], Aniko Gal[5,6], David Weaver[5], Rachele Rossi[2], Annarita Armaroli[2], Marika Pane[7], Rahul Phadke[3], Anna Sarkozy[3], Francesco Muntoni[3], Imelda Hughes[8], Antonella Cecconi[9], György Hajnóczky[5], Alice Donati[10], Eugenio Mercuri[7], Massimo Zeviani[11,]§, Alessandra Ferlini[2,8,*,]§, Daniele Ghezzi[1,*,]§

Supporting Information includes supplementary methods, 1 table and 13 figures.

SUPPLEMENTARY METHODS

Whole Exome Sequencing (WES)

Genomic DNA was extracted from peripheral blood by Nucleon Bacc3 kit (GE Healthcare Life Science). DNA samples were prepared for exome sequencing using the Nextera Exome Enrichment method from Illumina. Briefly, 50 ng of gDNA was tagmented using the Nextera transposome complex, generating adaptor ligated DNA fragments ready for enrichment. All samples were barcode/index labelled as a part of the tagmentation process. Samples were quantified using Picogreen measurements and the quality of each sample was assessed using the Agilent BioAnalyzer. Up to twelve samples were pooled together in equal quantities before capture. A bait library of >340,000, 95-

mer biotinylated probes was used to enrich a 62Mb region of the genome containing 201,121 coding exons (20,794 genes), UTR´s and ncRNA regions. The biotinylated probes were used to hybridize to the target sequences, followed by capture using streptavidin beads. The captured DNA was further amplifed by PCR and the quality of the pooled, enriched sequencing libraries was determined on the BioAnalyzer. Further quality control was done by sequencing each twelve sample pool on the Illumina MiSeq instrument, assessing optimal cluster densities, distribution of reads from each sample within the pool and other quality metrics such as insert size and duplications.

WES samples were sequenced on Illumina HiSeq 2500 instruments (4 lanes per pool), using TruSeq v4 cluster and SBS kits, respectively**.**

Alignment and variant calling.

Reads were aligned to NCBI Build 37 of the human reference sequence using Burrows-Wheeler Aligner (BWA) 0.5.9 [Li and Durbin, 2009]. Alignments were merged into a single BAM file and marked for duplicates using Picard 1.55 (http://picard.sourceforge.net/). Only non-duplicate reads were used for the downstream analyses. Variants were called using Genome Analysis Toolkit, (GenomeAnalysisTK) 1.2-29-g0acaf2d [McKenna et al., 2010], by applying base quality score recalibration, indel realignment and performing SNP and INDEL discovery and genotyping using standard hard filtering [DePristo et al., 2011]. Variants were annotated using SNPeff and Genome Analysis Toolkit 1.4-9-g1f1233b with only the highest-impact effect [McKenna et al., 2010]. Datasets were imported into Clinical Sequence Analyzer (CSA) for further datamining and variant discoveries. Nucleotide numbering reflects cDNA numbering with +1 corresponding to the A of the ATG translation initiation codon in the reference sequence, according to journal guidelines (www.hgvs.org/mutnomen). The initiation codon is codon 1. All variants reported have been submitted to LSDB (http://www.lovd.nl/MSTO1).

Mitochondrial DNA quantification

mtDNA content was evaluated by real-time PCR-based quantification (ABI7000 Real-Time PCR System) using two specific mtDNA probes and a standard, single-copy autosomal gene (RNaseP) for normalization [Gai et al., 2013].

Cell cultures

Skin fibroblasts from patients and controls subjects and Hela cells were cultured in either high glucose (25 mM) DMEM (Euroclone) or 5mM galactose, glucose-free DMEM medium supplemented with 10% FBS, 1% L-glutamine and 0.2% sodium pyruvate, in a 37°C incubator with 5% CO_2. All cell lines were mycoplasma-free. Fibroblasts were immortalized with pRNS-1 by transfection (Lipofectamine2000 Invitrogen) and selected by 100 μg/ml Geneticin G-418 (Gibco-Life Technologies).

Transcript analysis

Total RNA was extracted from skin fibroblasts pellets using the RNeasy Mini Kit (Qiagen, Milan, Italy) and 1 μg was used as a template for reverse transcriptase PCR (RT-PCR) to obtain full-length Cdna, using the GoTaq® 2-Step RT-qPCR System (Promega). *MSTO1* transcript was amplified through PCR and analysed by electrophoresis on 1% agarose gel in order to detect potential splicing alterations and PCR products were also sequenced in order to confirm genomic variants. *MSTO1* expression level was determined using reverse transcription quantitative qPCR with specific amplicons and SYBR-green chemistry.

Fluorescence microscopy

For visualization of the mitochondrial network, the mitochondrial fluorescent dye MitoTracker Red-CMXRos (Invitrogen) was added to the culture media at a final concentration of 50 nM for 30 min. and then images were acquired with a confocal microscope (Leica TSC-SP8). [Nasca et al., 2016]

Starting from a commercial clone encoding wild type *MSTO1* cDNA (MHS6278-202827318, GE Healthcare), we generated a customized pcDNA3.1 plasmid (Invitrogen) containing the wild-type

MSTO1 transcript, 3'-tagged with the sequence encoding an epitope of the influenza virus haemoagglutinin (HA) gene (MSTO1-HA) [Ghezzi et al., 2009]. For overexpression studies, cells were transfected using Lipofectamine2000 (Invitrogen): 1) HeLa and COS7 cells were transiently transfected with MSTO1-HA; 2) fibroblasts were transiently co-transfected with two clones encoding MSTO1-HA and a GFP targeted to mitochondria (mtGFP) [Weber et al., 2013; Perli et al., 2014].

For visualization of MSTO1, after fixation (Paraformaldehyde 4%, 15 min.) and permeabilization (Triton X-100 0.2%, 5 min.), cells were incubated with a mouse anti-HA (12CA5, Roche) antibody, followed by a fluorescently labeled secondary antibody (Alexa Fluor 488, Invitrogen) [Ghezzi et al., 2009]. The nuclear dye TO-PRO-3 (Invitrogen) was added at a concentration of 1µM during the incubation with the secondary antibody.

For studies on mitochondrial dynamics, imaging was performed as previously described [Weaver et al., 2014; Eisner et al., 2014]. Briefly, cells were incubated in a 0.25% BSA containing extracellular medium (ECM) consisting of 121 mM NaCl, 5 mM $NaHCO_3$, 4.7 mM KCl, 1.2 mM KH_2PO4, 1.2 mM $MgSO_4$, 2 mM $CaCl_2$, 10 mM glucose, and 10 mM Na-Hepes, pH 7.4, at 37°C. The cells were co-transfected with cDNAs encoding mtDsRed1 and mitochondrial matrix-targeted photoactivatable GFP (mtPA-GFP). Recordings of mtPA-GFP and mtDsRed (512 × 512 pixels) were performed using 488 nm and 561 nm laser lines at 0.25 s^{-1} data acquisition frequency using the LSM780 microscope. To photoactivate PA-GFP in 2P mode, a pulsed laser system (760 nm, Chameleon; Coherent, Inc.) was applied. Using confocal microscopy, time series of fluorescence images were recorded and 25 μm^2 square-shaped areas were illuminated by a pulsed laser to photoactivate mtPA-GFP [Eisner et al., 2014; Weaver et al., 2014], which is a soluble protein that show rapid diffusion [Partikian et al., 1998]. Mitochondrial matrix continuity and connectivity is unveiled by the diffusion of the photoactivated mtPA-GFP to the regions outside the 2P illuminated area. To quantify connectivity, the time course of

the ratio of $F_{mtPA-GFP}$ ($G-G_{base}/G_{max}-G_{base}$) was calculated for the region of photoactivation (RPA). Image analysis was done using either Spectralyzer or Zen2010.

Analysis of mitochondrial matrix continuity

Mitochondrial matrix continuity was evaluated by two different approaches. Spreading of PA-GFP from the area of photoactivation was evaluated by masking the 5×5 μm areas and quantifying the time-dependent decay in the fluorescence intensity. The images were thresholded to reach a 0.1% noise-to-signal ratio. Then the pixels of the photoactivated areas were subtracted and the PA-GFP–positive pixels were counted. Image analysis was performed in Spectralyzer imaging software or in Zen2010. Only cells with the same number of photoactivated areas and no focus loss were compared.

Peroxisomal and Picogreen staining

For visualization of peroxisomes, after fixation and permeabilization, cells were incubated with an anti-PMP70 (ABT12, Millipore) antibody, followed by a fluorescently labeled secondary antibody (Alexa Fluor 488, Invitrogen) [Nasca et al. 2016].

For visualization of mitochondrial and nuclear DNA, cells were incubated with Picogreen (Invitrogen) at a concentration of 3μl/ml, for 1h at 37°C; then images were acquired with a confocal microscope (Leica TSC-SP8).

Cell fractionation

Mitochondrial isolation and subcellular fractionation were performed as previously described [Fernandez-Vizarra et al., 2002] either in fibroblasts or Hela cells.

Western blot analysis

Cells were pelleted and solubilized in RIPA buffer with protease inhibitors; 30-50 μg of protein was loaded for each sample in 10% denaturing sodium-dodecyl sulphatepolyacrylamide gel electrophoresis (SDS-PAGE). Polyclonal antibodies against MSTO1 (SAB2700296, Sigma), DNM1L/DRP1 (D6C7,

#8570, Cell Signaling), and monoclonal antibodies against GAPDH (#MAB374, Millipore), and cII subunit SDHA (ab14715, Mitosciences) were used.

Supp. Table S1: prediction of subcellular localization

Tools	Prediction
**Bacello**	Localization: Cytoplasm
**Psort**	Localization: 47.8 %: cytoplasmic,<br>26.1 %: mitochondrial
**Mitoprot**	PROBABILITY of export to mitochondria: 0.0286
**TargetP**	PROBABILITY of export to mitochondria: 0.132

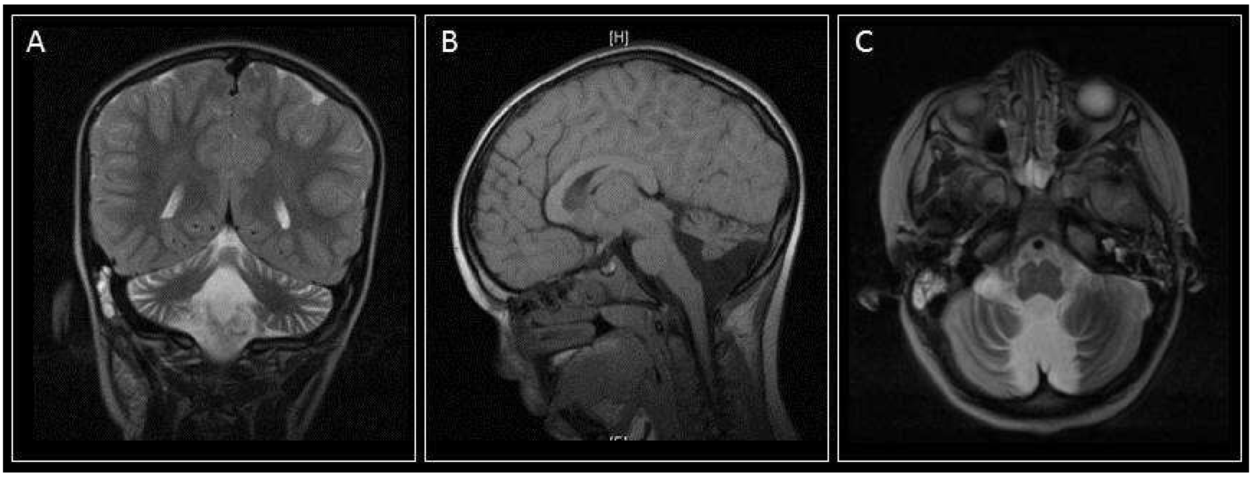

Supp. Figure S1: Brain MRI images of the patient B at age 3 ½ years.

A. T2-weighted coronal image;

B. T1-weighted sagittal image;

C. T2-weighted transverse image.

Please note cerebellar hypoplasia/atrophy involving the vermis and the cerebellar hemispheres, with the pons being relatively preserved.

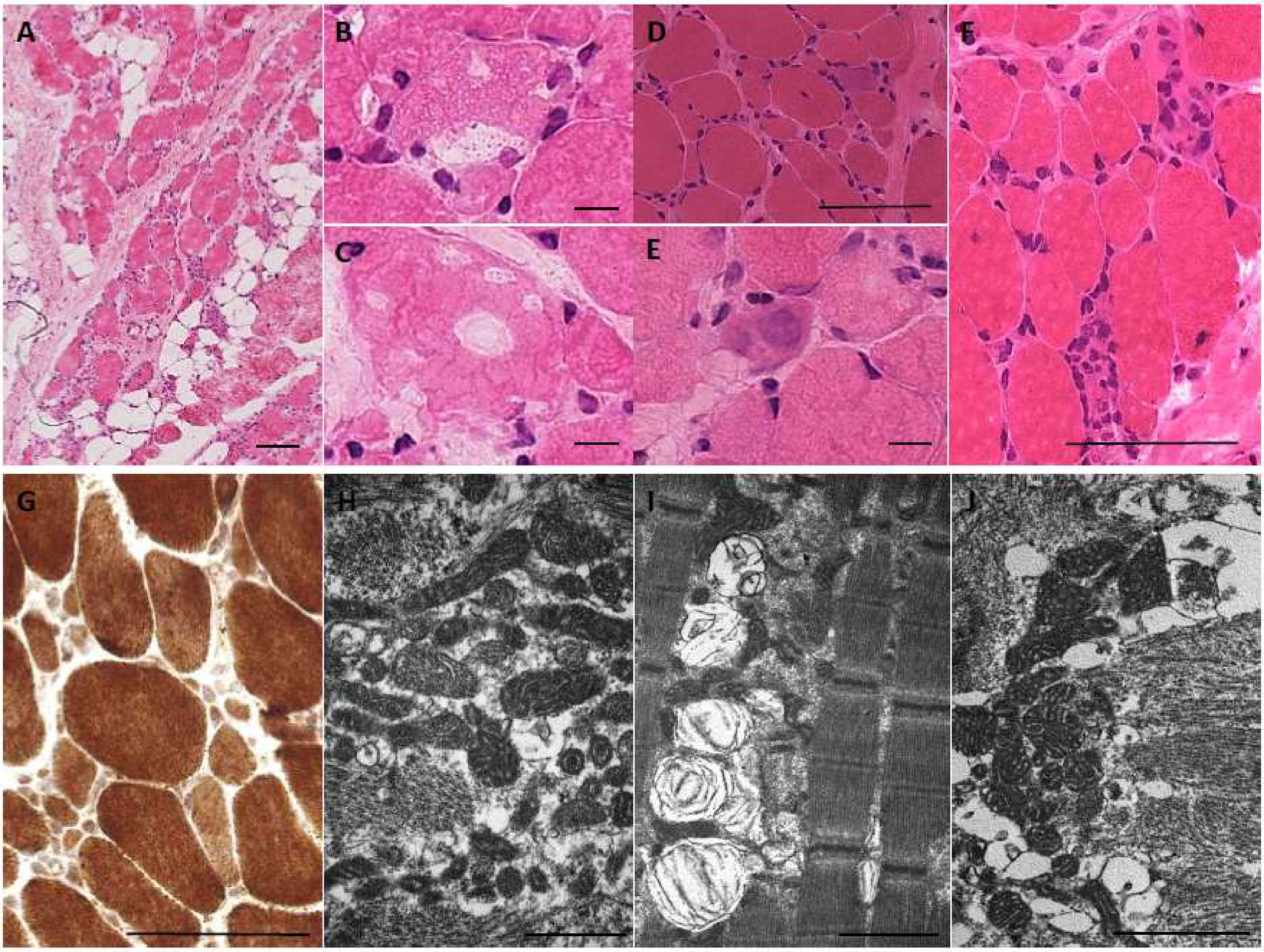

Supp. Figure S2: Histochemical analysis of the patient B's muscle biopsy

Right thigh muscle biopsy performed at 2 years. Haematoxylin and Eosin stained sections showed mild-to-moderate dystrophic changes with patchy fatty infiltration and fibrosis within fascicles (A), increased variation in fibre size (A, D, F), fibres with non-rimmed, often empty vacuoles (B, C), scattered basophilic regenerating fibres (D, E) and necrotic fibres infiltrated by macrophages (F). No COX-negative fibres are seen in the sequential COX-SDH reaction (G). Ultrastructural examination showed aggregates of mitochondria with varied size, shape and abnormal circular or concentric cristae (H). Mitochondria were often seen undergoing vacuolar degeneration (I, J).

Scale bar: A, D, E, G = 100 µm, B, C, E = 10 µm, H= 0.5 µm, I, J = 1 µm

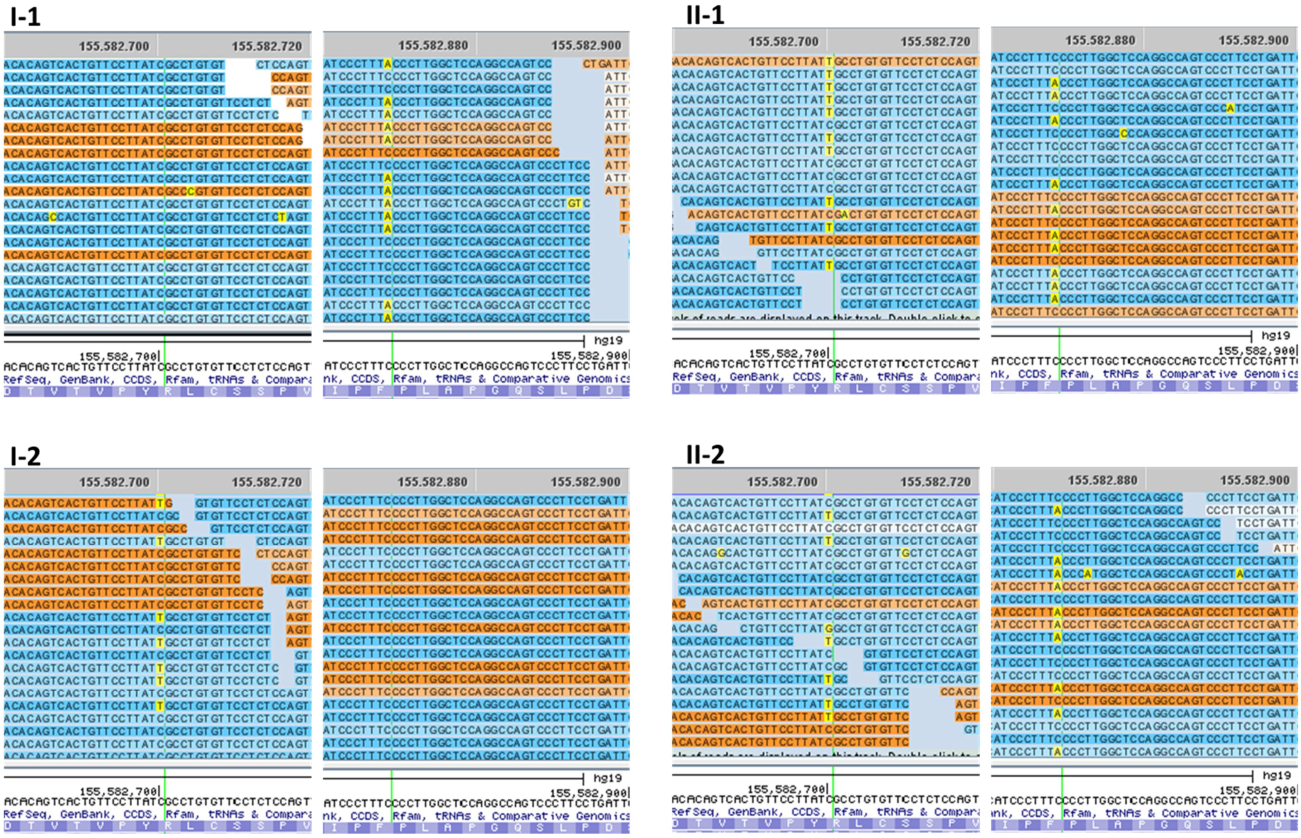

Supp. Figure S3: NGS results from family A

Snapshot of the sequencing reads covering the compound heterozygous mutations in the affected daughters (II-1, II-2) and the healthy parents (I-1, I-2) of family A. The variation is highlighted by the yellow rectangles. Forward and reverse reads are indicated respectively by blue and orange colors.

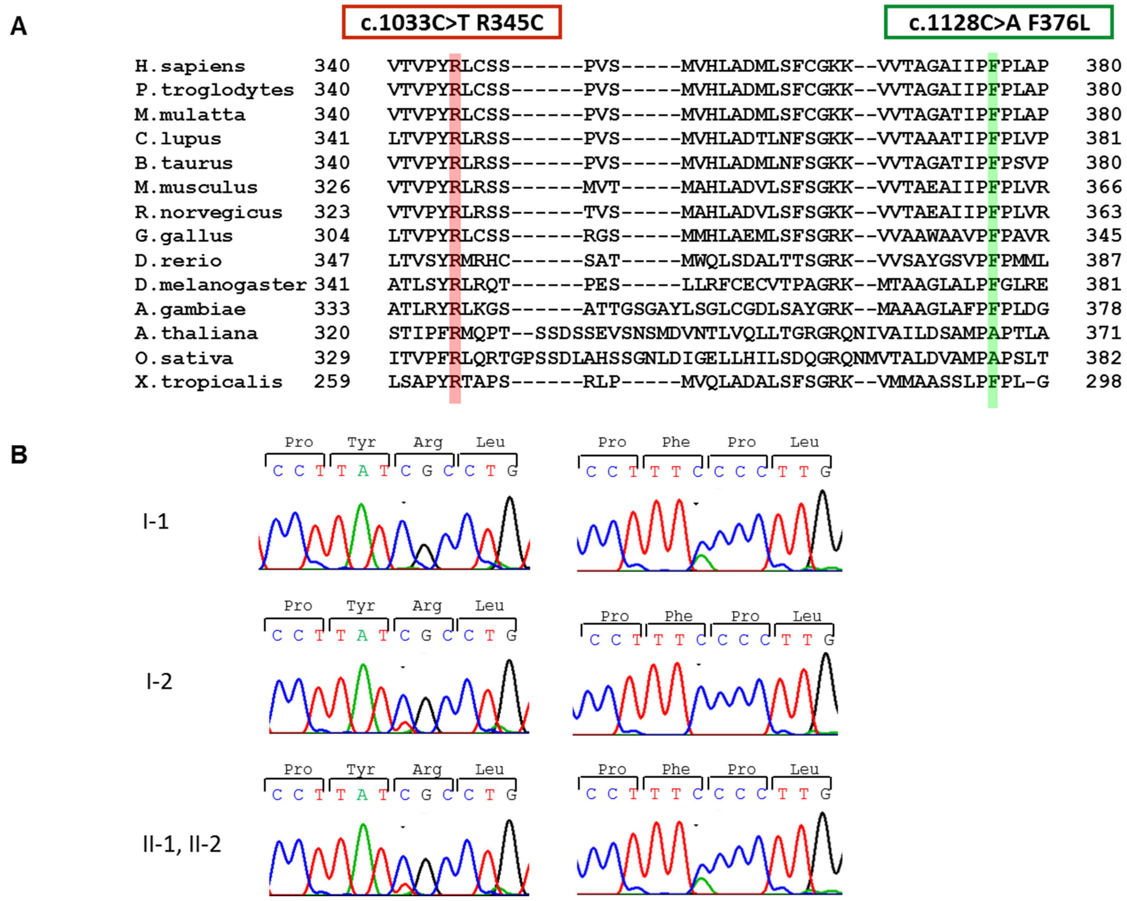

Supp. Figure S4: Variant analyses in family A

A. Phylogenetic conservation of the amino acid residues affected by the missense mutations identified in the patients A1 and A2.

B. Electropherograms of the genomic regions harboring the *MSTO1* mutations, in members of family A: the father (I-1), the mother (I-2), the affected siblings (II-1, II-2).

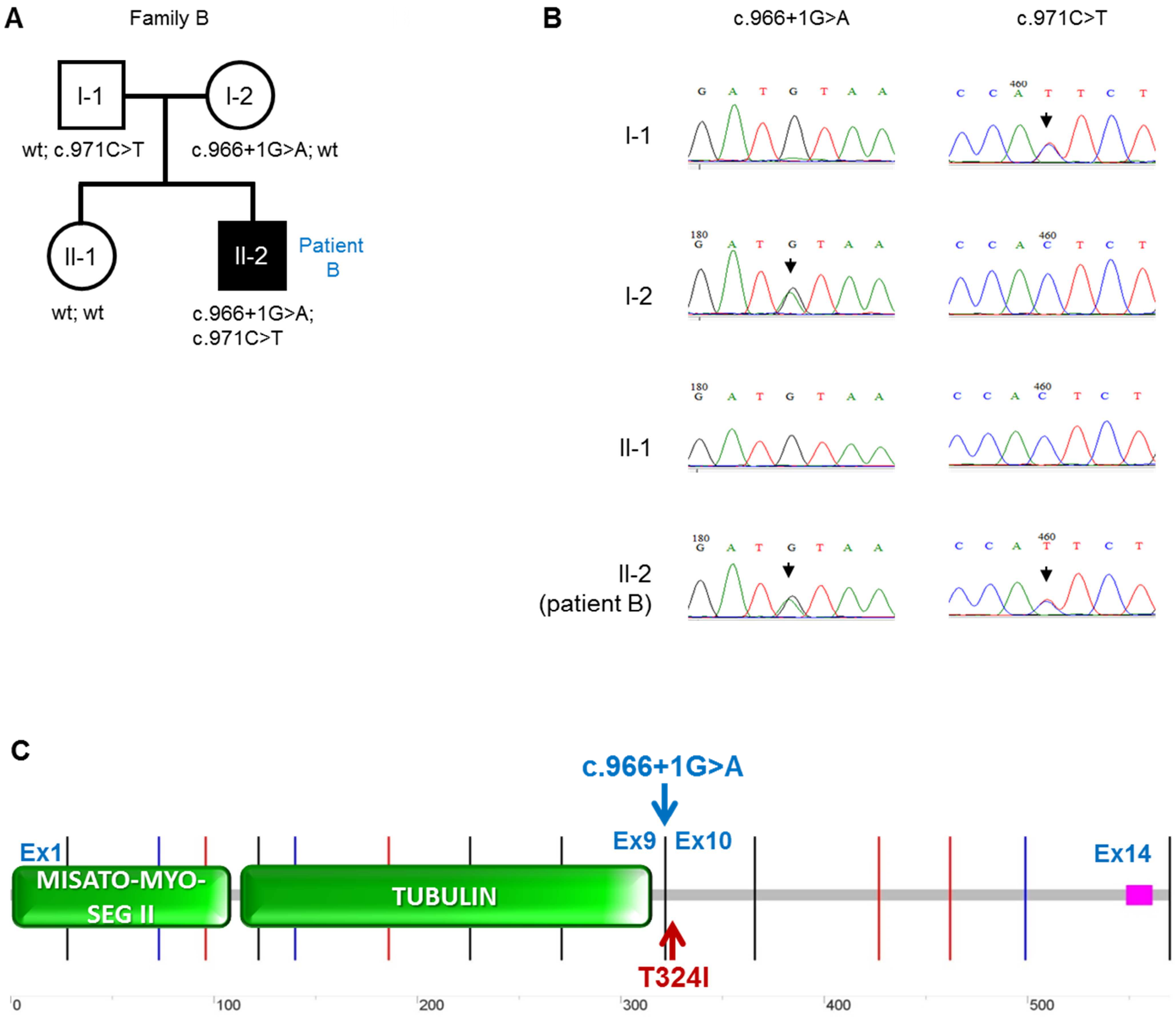

Supp. Figure S5: Variant analysis in patient B

A. Pedigree of the family B with the identified *MSTO1* variants. Black symbol indicates the affected subject. wt= wild-type sequence.

B. Electropherograms of the genomic regions harboring the *MSTO1* mutations, in members of family B: the father (I-1), the mother (I-2), the healthy affected sibling (II-1) and patient B (II-2).

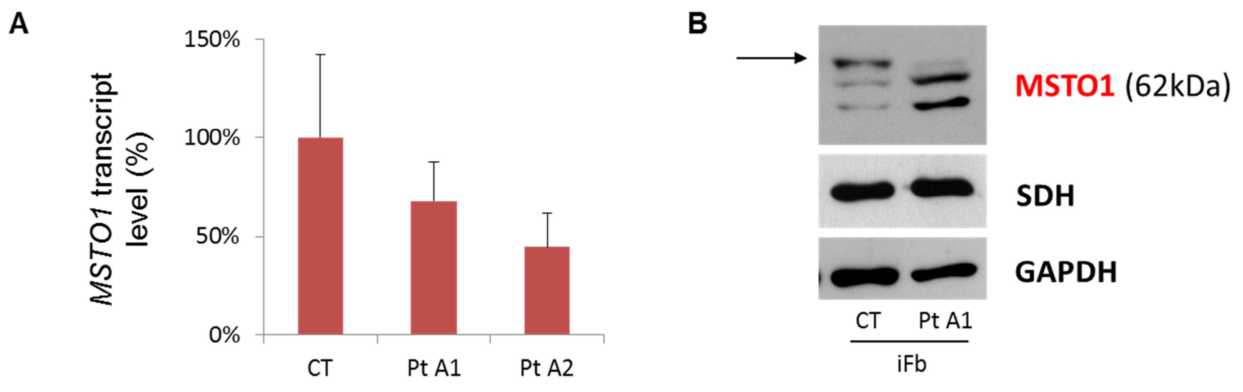

Supp. Figure S6: Variant analyses

A. *MSTO1* transcript levels in patients (Pt A1 and A2) fibroblasts compared to controls (CT). . Data are represented as mean + SD of four independent experiments and reported as percentage. In each experiment *MSTO1* values were normalized to both *GAPDH* and *ACTB* expression levels. Pt A1 vs CT: p=0.07; Pt A2 vs CT: p=0.004 (unpaired two-tailed t-test).

B. MSTO1 protein amount in immortalized fibroblasts (iFb) from patient A1 (Pt A1) and control (CT), obtained using an anti-MSTO1 antibody. Anti-GAPDH and anti-SDH antibodies were used as a loading controls.

A

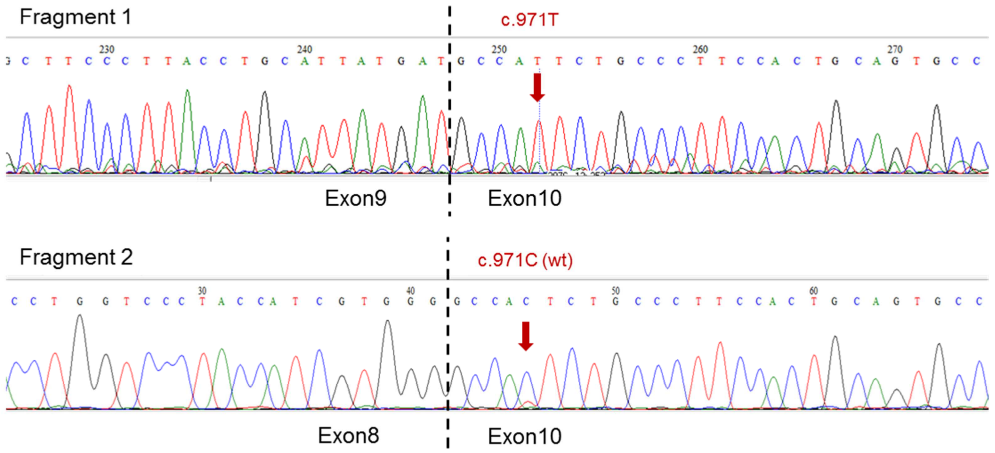

B

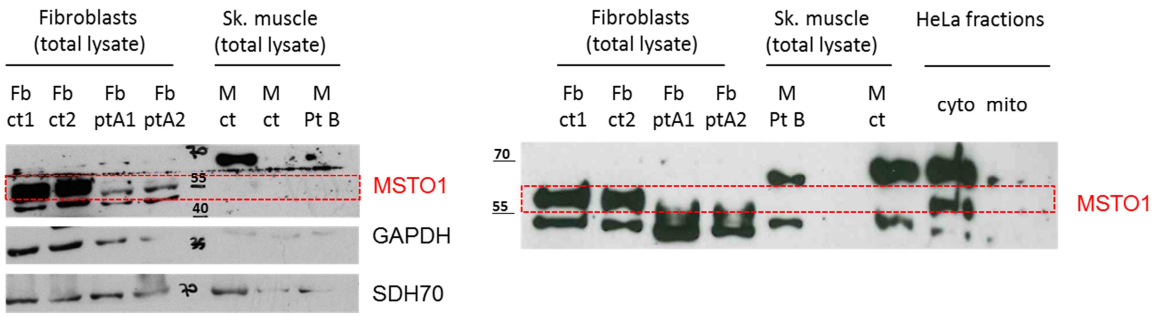

Supp. Figure S7: Functional studies on muscle from patient B

A. Electropherograms of the two fragments obtained by PCR amplification of cDNA obtained from retrotranscribed RNA of patient B's muscle. Sequence of fragment 1 (with a molecular weight as the control) revealed the presence of the c.971C>T (as homozygous) while sequence of fragment 2 (with a molecular weight lower than control) showed the skipping of exon 9.

B. Immunoblot analysis in specimens from patients (fibroblasts from patients A1 and A2; skeletal muscle from patient B) and controls. Note that in muscle samples, no immunoreactive signal was observed at the expected molecular weight while a clear band was present in control fibroblasts and in the cytosolic fraction from HeLa cells.

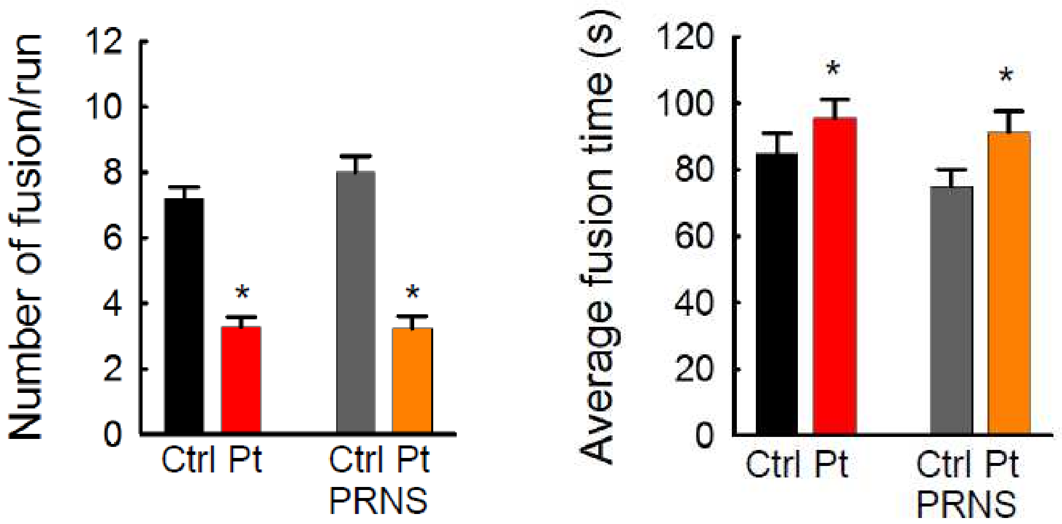

Supp. Figure S8: Mitochondrial fusion events in fibroblasts

A. Rates of fusion events in primary and immortalized fibroblasts from patient A1 (Pt). Reciprocal spreading of mtPA-GFP and mtDsRed among mitochondria that were not continuous at the time of mtPA-GFP photoactivation was sought. The fusion events were quantitatively analyzed by the progression of the distribution of mtPA-GFP fluorescence between the images collected in the first 8 min after photoactivation. (Number of fusions: CT N=186; MSTO Pt N=95; iCT N=178; iPt N=68 from 3 experiments per each cells). Stars indicate the significantly differences ($p<0.05$) (Pt: 3.27±0.3 fusion/run; iPt: 3.24±0.37 fusion/run; CT: 7.21±0.33 fusion/run, iCT: 8.0±0.41 fusion/run).

B. Average duration of fusion events which were followed by fission during the 8 min recording time. Stars indicate significantly differences ($p<0.05$).

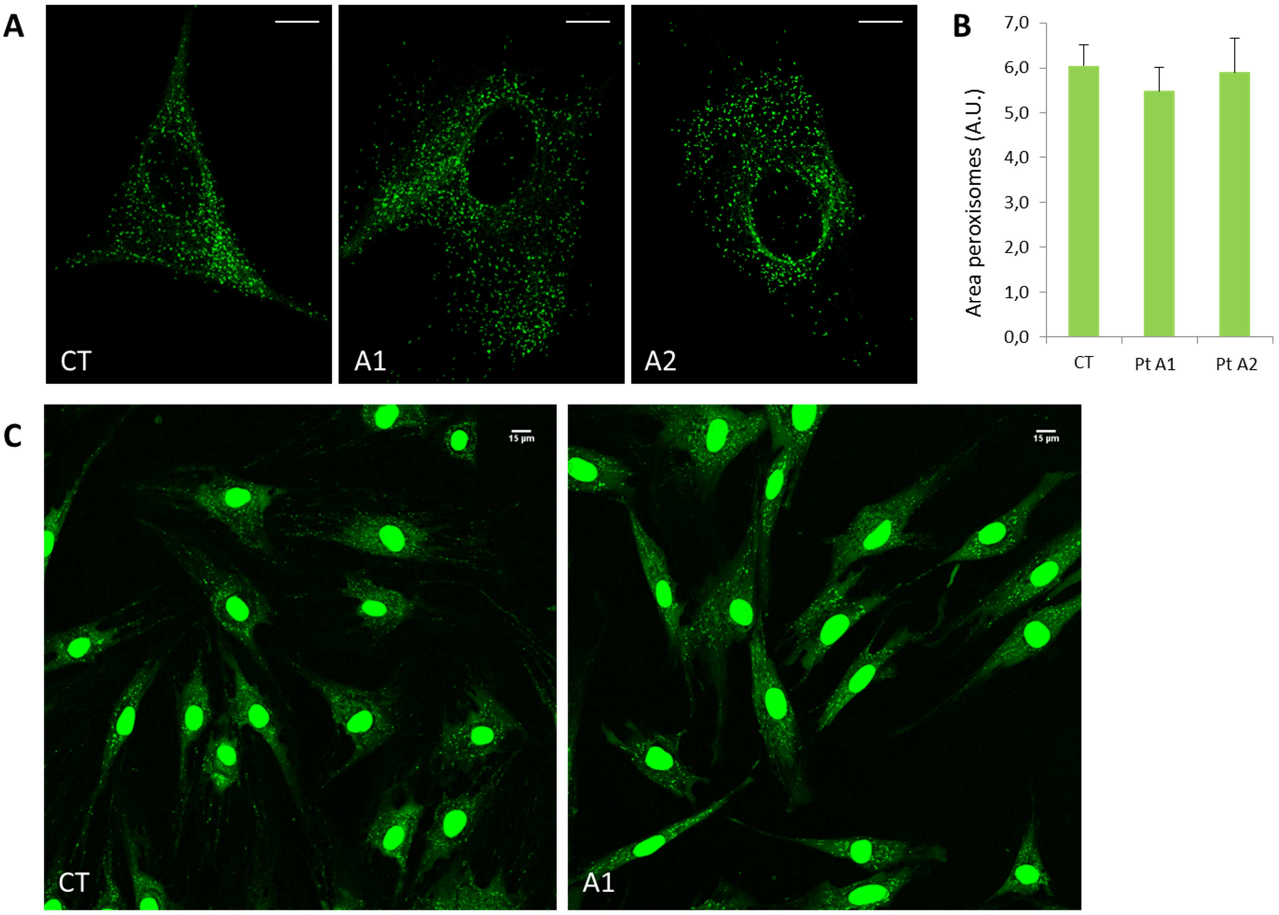

Supp. Figure S9: Peroxisomal and Picogreen staining

A. Representative images of the peroxisomal staining obtained with anti-PMP70 antibody in fibroblasts from control (CT) and patients (A1, A2). Scale bars: 10 µm. The shape factor was similar in all samples (CT: 0.94±0.01; II-1: 0.95±0.01; II-2: 0.94±0.01).

B. Analysis of the mean area of the peroxisomal staining in fibroblasts from CT, A1 and A2.

C. Picogreen staining for visualization of mitochondrial and nuclear DNA, in fibroblasts from a control (CT) and patient A1. No evident alteration was observed in mitochondrial nucleoid distribution (e.g. perinuclear clustering) or in nuclear morphology (e.g. nuclear fragmentation). Scale bars: 15 µm.

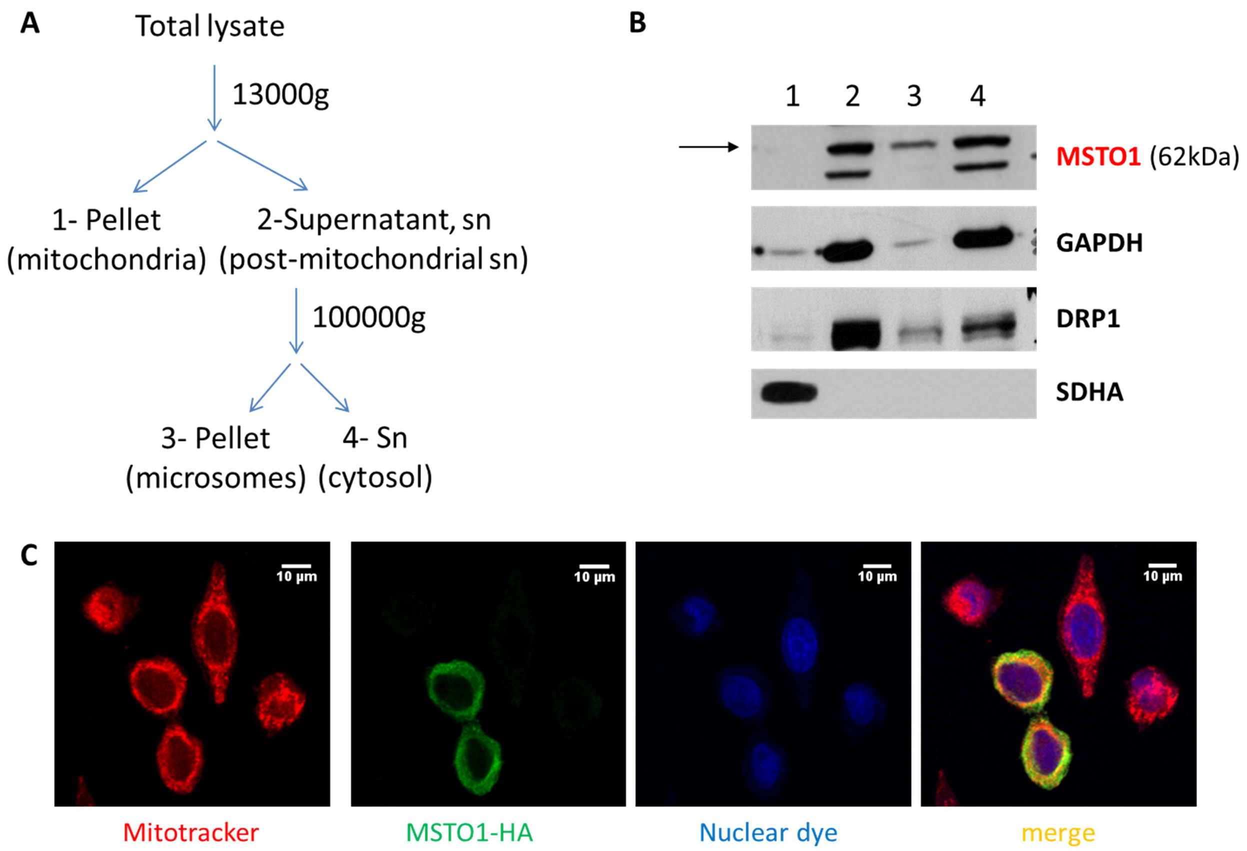

Supp. Figure S10: MSTO1 subcellular localization

A. Schematic representation of the subcellular fractionation protocol.

B. Immunoblot analysis of samples obtained from HeLa total cell lysate according to the procedure described above (panel A), using α-MSTO1, α-GAPDH (cytosolic protein), α-DNM1L (cytosolic protein with a role in mitodynamics), α-SDHA (mitochondrial protein) antibodies.

C. Immunofluorescence images of HeLa cells transfected with MSTO1-HA. The red signal corresponds to Mitotracker, the green signal corresponds to the anti-HA immunoreaction, the blue signal corresponds to the nuclear dye TO-PRO-3. Scale bars: 10 µm.

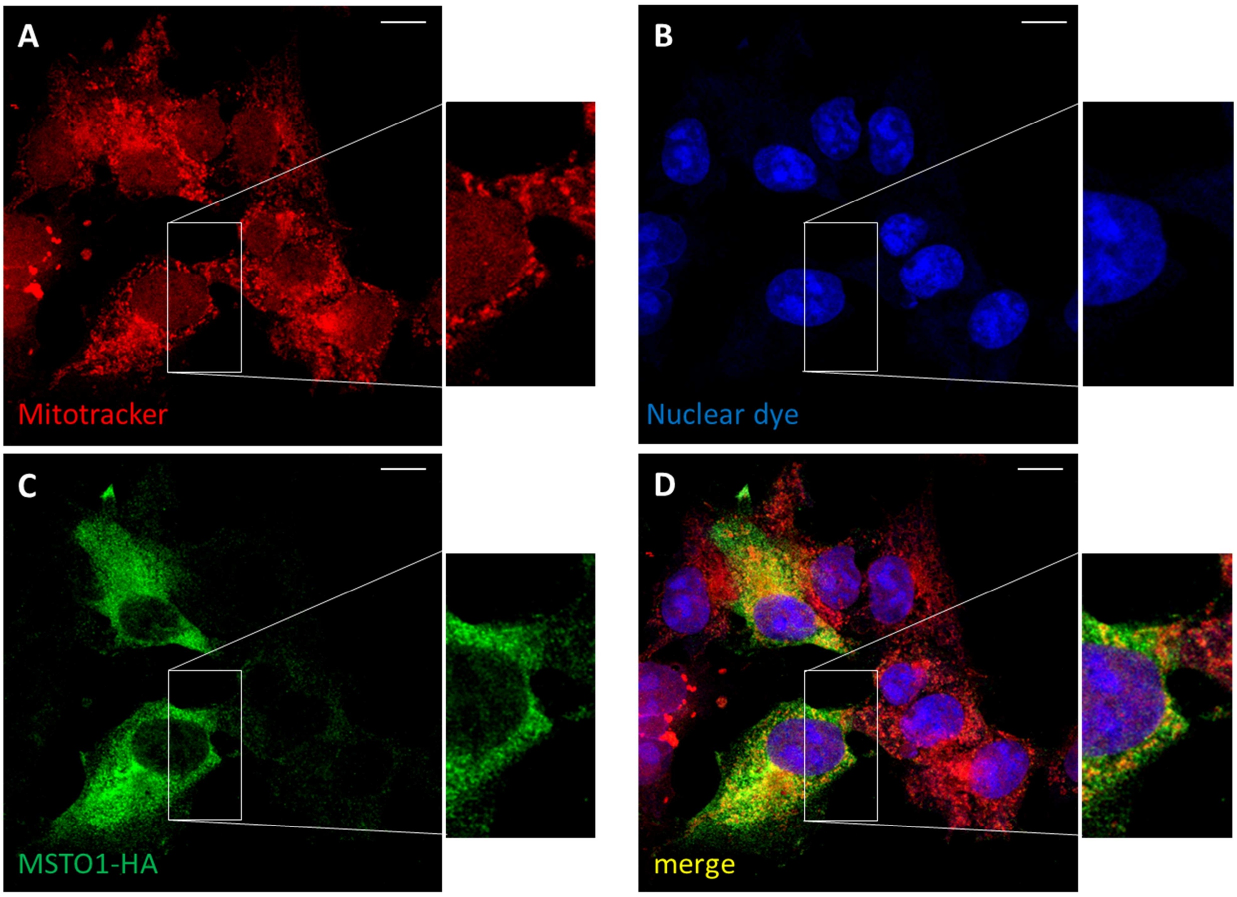

Supp. Figure S11: Immunofluorescence studies in COS7 cells

Immunofluorescence images of COS7 cells transfected with MSTO1-HA, taken 24 hours after transfection. In A. the red signal corresponding to Mitotracker red; in B. the green signal corresponding to the anti-HA immunoreaction; in C. the blue signal corresponding to the nuclear dye TO-PRO-3; in D. the merged image with the overlay of red, green and blue channels. Scale bars: 10 µm.

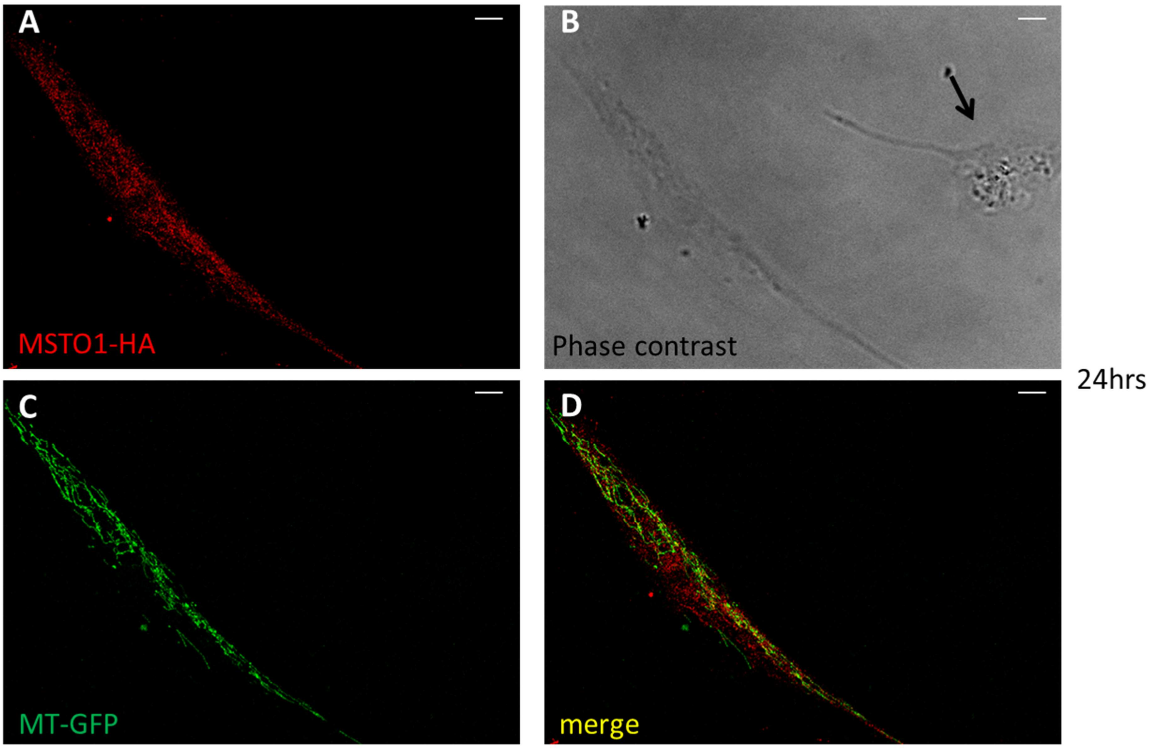

Supp. Figure S12: Immunofluorescence studies in fibroblasts (fixed cells)

Immunofluorescence images of fibroblasts from patient A1, co-transfected with wild type MSTO1-HA and a mitochondrial GFP (MT-GFP), fixed and stained 24hours after transfection in order to demonstrate the contemporary expression of both vectors. In A. the red signal corresponding to the anti-HA immunoreaction; in B. the green signal corresponding to MT-GFP; in C. a phase-contrast image (the arrow indicates a non-transfected cell); in D. the merged image with the overlay of red and green channels. Scale bars: 15 µm.

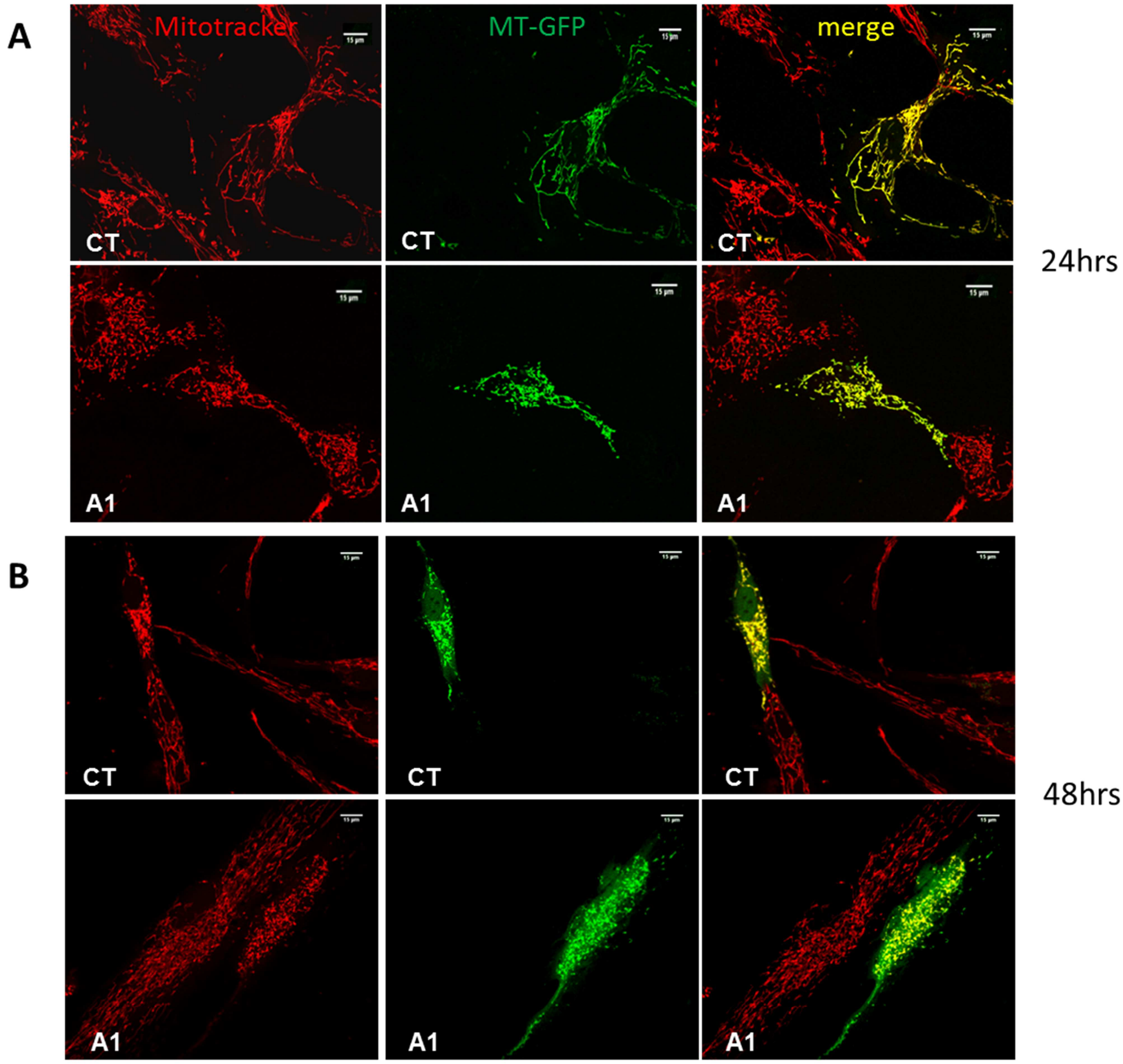

Supp. Figure S13: Immunofluorescence studies in fibroblasts (live cells)

Immunofluorescence images of fibroblasts from patient A1 and control (CT), co-transfected with wild type MSTO1-HA and a mitochondrial GFP (MT-GFP), taken 24hours (panel A) and 48 hours (panel B) after transfection. The red signal corresponds to Mitotracker red, the green signal corresponds to the MT-GFP. Scale bars: 15 µm.
